# Exploring preconception signatures of metabolites in mothers with gestational diabetes mellitus using a non-targeted approach

**DOI:** 10.1186/s12916-023-02819-5

**Published:** 2023-03-16

**Authors:** Ling-Jun Li, Ximeng Wang, Yap Seng Chong, Jerry Kok Yen Chan, Kok Hian Tan, Johan G. Eriksson, Zhongwei Huang, Mohammad L. Rahman, Liang Cui, Cuilin Zhang

**Affiliations:** 1 Global Centre for Asian Women’s Health, Yong Loo Lin School of Medicine, National University of Singapore, Singapore, Singapore; 2 Department of Obstetrics and Gynaecology, Yong Loo Lin School of Medicine, National University of Singapore, Singapore, Singapore; 3 NUS Bia-Echo Asia Centre for Reproductive Longevity and Equality (ACRLE), Yong Loo Lin School of Medicine, National University of Singapore, Singapore, Singapore; 4 Global Health Research Center, Guangdong Provincial People’s Hospital, Guangdong Academy of Medical Sciences, Southern Medical University, Guangzhou, China; 5 Guangdong Cardiovascular Institute, Guangdong Provincial People’s Hospital, Guangdong Academy of Medical Sciences, Southern Medical University, Guangzhou, China; 6 Duke-NUS Medical School, Singapore, Singapore; 7 KK Women’s and Children’s Hospital, Singapore, Singapore; 8 Department of General Practice and Primary Health Care, University of Helsinki and Helsinki University Hospital, Helsinki, Finland; 9 Folkhälsan Research Center, Helsinki, Finland; 10 Agency for Science, Technology and Research (A*STAR), Singapore Institute for Clinical Sciences (SICS), Singapore, Singapore; 11 Institute of Molecular and Cell Biology, Agency of Science, Technology and Research, Singapore, Singapore; 12 Occupational and Environmental Epidemiology Branch, Division of Cancer Epidemiology and Genetics, National Cancer Institute, National Institutes of Health, Rockville, MD USA; 13 Antimicrobial Resistance Interdisciplinary Research Group, Singapore-MIT Alliance for Research and Technology, Singapore, Singapore

**Keywords:** Preconception, Non-targeted metabolomics, Metabolites, Phosphatidylethanolamines, Lipids, Gestational diabetes mellitus, Prediction

## Abstract

**Background:**

Metabolomic changes during pregnancy have been suggested to underlie the etiology of gestational diabetes mellitus (GDM). However, research on metabolites during preconception is lacking. Therefore, this study aimed to investigate distinctive metabolites during the preconception phase between GDM and non-GDM controls in a nested case–control study in Singapore.

**Methods:**

Within a Singapore preconception cohort, we included 33 Chinese pregnant women diagnosed with GDM according to the IADPSG criteria between 24 and 28 weeks of gestation. We then matched them with 33 non-GDM Chinese women by age and pre-pregnancy body mass index (ppBMI) within the same cohort. We performed a non-targeted metabolomics approach using fasting serum samples collected within 12 months prior to conception. We used generalized linear mixed model to identify metabolites associated with GDM at preconception after adjusting for maternal age and ppBMI. After annotation and multiple testing, we explored the additional predictive value of novel signatures of preconception metabolites in terms of GDM diagnosis.

**Results:**

A total of 57 metabolites were significantly associated with GDM, and eight phosphatidylethanolamines were annotated using HMDB. After multiple testing corrections and sensitivity analysis, phosphatidylethanolamines 36:4 (mean difference *β*: 0.07; 95% CI: 0.02, 0.11) and 38:6 (*β*: 0.06; 0.004, 0.11) remained significantly higher in GDM subjects, compared with non-GDM controls. With all preconception signals of phosphatidylethanolamines in addition to traditional risk factors (e.g., maternal age and ppBMI), the predictive value measured by area under the curve (AUC) increased from 0.620 to 0.843.

**Conclusions:**

Our data identified distinctive signatures of GDM-associated preconception phosphatidylethanolamines, which is of potential value to understand the etiology of GDM as early as in the preconception phase. Future studies with larger sample sizes among alternative populations are warranted to validate the associations of these signatures of metabolites and their predictive value in GDM.

**Supplementary Information:**

The online version contains supplementary material available at 10.1186/s12916-023-02819-5.

## Background

Metabolic alterations in a healthy pregnancy include a 30% increment in basal endogenous glucose production by late pregnancy, primarily by hepatic function [1]. In order to maintain euglycemia, circulating fasting glucose concentrations decrease during pregnancy mainly due to an increase in plasma volume in early pregnancy and an increase in glucose utilization in later gestation by the fetoplacental unit [2, 3]. Women with disruption during such metabolic adaptation during any time of pregnancy, such as reduced peripheral insulin sensitivity (e.g., reduced glucose uptake in skeletal muscle and adipose tissue, adverse amino acid, and lipid metabolism) [1] and diminished pancreatic β-cell reserve [4], could develop hyperglycemia also known as gestational diabetes mellitus (GDM).

GDM affects from 1% to > 30% of pregnancies worldwide and is exceptionally prevalent in Asian populations such as Saudi Arabia, India, and Singapore [5]. GDM is also important for public health awareness for two reasons. To begin with, GDM increases the risk of pregnancy complications for both mothers and offspring [3]. Subsequently, a GDM diagnosis identifies populations at risk (i.e., women and their offspring) of obesity, diabetes, and premature cardiovascular disease in the long run [3, 6, 7]. The etiology of GDM remains unclear, even though its involvement with maternal obesity, inflammation, and oxidative stress-mediated insulin resistance has been widely suggested [3, 8].

Metabolomics approach, defined as the study of global metabolite profiles in bio-samples under a given set of conditions, has been beneficial for assessing cardiometabolic conditions [9]. Emerging evidence rooted in epidemiological studies has also investigated plasma, serum, and even urine metabolite profiles during pregnancy that significantly differed between women with and without GDM [8, 10–15]. However, the findings in metabolite panels were inconsistent, and later assessment in pregnancy was subjective to reverse causality [9, 16]. In addition, metabolic disruption as early as the preconception phase could help in understanding the etiology of GDM, which has been lacking in current literature. Therefore, we aimed to investigate distinctive metabolites during the preconception phase between GDM and non-GDM controls using a non-targeted approach, in a nested case–control study among homogenous Chinese pregnant subjects in Singapore.

## Methods

### Study population and study design

Within the Singapore PREconception Study of long-Term maternal and child Outcomes (SPRESTO), a preconception and pregnancy cohort conducted from February 2015 to October 2017, we recruited Chinese, Malay, and Indian women without type 1 or type 2 diabetes, aged 18–45 years, and planning to conceive within 12 months. We published the cohort profile earlier and described the study objective and protocol in detail [17]. The study was conducted according to the guidelines under the Declaration of Helsinki and approved by the SingHealth Centralized Institute Review Board (2014/629/D).

### GDM diagnosis

Among 376 singleton live births, we ascertained 33 Chinese pregnant women with GDM according to the International Association of Diabetes and Pregnancy Study (IADPSG) criteria between 24 and 28 weeks of gestation [18]. We then matched them at a 1:1 ratio with 33 non-GDM Chinese women by age (± 2 years) and categories of pre-pregnancy body mass index (ppBMI) (underweight [< 18.5 kg/m^2^], normal weight [18.5–22.9 kg/m^2^], overweight [23.0–27.4 kg/m^2^], and obese [≥ 27.5 kg/m^2^]) [17]. We performed blood collection among all participants during the preconception phase (within 12 months prior to conception).

### Non-targeted metabolomics approach

Non-targeted metabolomics analysis was performed using liquid chromatography-mass spectrometry (LC–MS), as described in accordance with standard procedures [19, 20]. Upon enrolment during the preconception phase, we collected fasting serum samples from all participants and stored biospecimens at – 80 °C until thawing them immediately before assay. Briefly, fasting serum samples were processed with methanol or butanol/methanol precipitation for polar or non-polar metabolite analysis and were run in the same batch. Quality control samples were prepared by mixing equal amounts from all the samples and analyzed after every ten samples to monitor the stability of the system. We then reported the intra-coefficient of variation (CV) accordingly. LC–MS analysis was conducted using Agilent 1290 ultrahigh pressure liquid chromatography system equipped with a 6550 quadrupole time-of-flight mass detector managed by a MassHunter workstation. Mass data were collected between m/z 100 and 1000 Da at a rate of two scans per second. The electrospray ionization mass spectra were acquired in both positive and negative ion mode. Two reference masses were continuously infused to the system to allow constant mass correction during the run: m/z 121.0509 (C_5_H_4_N_4_) and m/z 922.0098 (C_18_H_18_O_6_N_3_P_3_F_24_). Raw spectrometric data were analyzed by the Agilent MassHunter Qualitative Analysis software. The molecular features characterized by retention time, chromatographic peak intensity and accurate mass were obtained using Agilent MassHunter Mass Profiler Professional software. A total of 5263 features extracted from both positive and negative ion modes were used for subsequent statistical analyses. All these features have an intensity ≥ 20,000 counts (approximately three times the detection limit of our LC–MS instrument) and were found in at least 80% of the samples in either GDM or non-GDM groups.

### Covariates

During recruitment, trained research coordinators conducted in-person interviews and measured the participants’ weight, height, and blood pressure at the study clinic. Covariates collected via questionnaires at study entry included socio-demographic factors, health history, menstrual characteristics, and lifestyle behaviors. In detail, they were maternal age, parity (nulliparous vs. parous), pre-pregnancy BMI, family history of diabetes, smoking (never vs. past or current), alcohol intake (never vs. past or current), micronutrient supplements intake in the past 3 months (yes vs. no). In addition, a 2-h 75 g two-time point oral glucose tolerance test (OGTT) was performed at the clinic during the first preconception visit. Preconception prediabetes was diagnosed based on fasting glucose concentration at ≥ 6.1 mmol/L and/or 2-h glucose concentration at ≥ 7.8 mmol/L, according to World Health Organization (WHO) guidelines [21].

### Statistical analyses

We performed the following steps for our statistical analyses. Step 1, we compared baseline characteristics between GDM and non-GDM controls using either generalized linear mixed model (GLMM) or generalized estimation equation (GEE) to account for 1:1 ratio matching effect. Step 2, we applied Student’s *t*-test to identify a pool of metabolite candidates, from which we further used GLMM to identify signatures of metabolites with adjustment of maternal age and ppBMI and accounting for the case–control matching effect. Step 3, upon GLMM identification, we performed annotation via Human Metabolite Database (HMDB) and pathway analysis via the KEGG database and assessed the correlation among all signatures of metabolites using the spearman rank correlation. Step 4, we compared the means and standard errors of all annotated metabolites after log-transformation their peak area between GDM and non-GDM controls using GLMM model and indicated regression estimate in mean difference between GDM and non-GDM subjects for each signature of metabolite identified. Due to the exploratory nature of our study, we corrected multiple testing using the false discovery rate (FDR) approach [22] and conducted sensitivity analysis by additionally adjusting for preconception diabetes according to WHO 2015 criteria [21], family history of diabetes, and parity. Step 5, all annotated metabolites from the preconception phase were presented in box plots, including median and inter-quartile range (IQR). Step 6, we performed the receiver operating characteristic (ROC) curve to calculate the area under the curve (AUC) value for GDM using annotated metabolites and traditional risks (e.g., maternal age, ppBMI) at the preconception phase.

In the descriptive table, we expressed all maternal characteristics data as median with IQR or mean with standard deviation (SD) when appropriate. We conducted all statistical analyses using the SIMCA 13.2 (Umetrics, Umea, Sweden), *MetaboAnalyst* *(Version 4.0)*, and *R* Software *(Version 3.5.0)*. We reported regression estimates in mean difference with 95% confidence interval (CI) after log-transformation of all signatures of metabolites and deemed significance at *p*-value (2-sided) less than 0.05.

## Results

No significant difference in baseline maternal characteristics were identified between GDM cases and matched controls (Table 1). A total of 57 metabolites were significantly associated with GDM. Nine were further successfully annotated. Since two were duplicated, a total of eight phosphatidylethanolamines were identified using HMDB, including 34:1, 34:2, 36:2, 36:4, 38:4, 38:5, 38:6, and 40:6. All of them were normalized after log-transformation in peak area and highly correlated with each other using the spearman rank correlation (Additional file 1: Tab. S1).

**Table 1:** Preconception maternal characteristics between GDM and non-GDM matching controls by maternal age, ethnicity, and pre-pregnancy body mass index category

Maternal characteristics before pregnancy	GDM subjects (*N* = 33)	Non-GDM subjects (*N* = 33)	p*
	Mean (SD) or *N* (%)	Mean (SD) or *N* (%)	
Age, years	30.09 (2.52)	30.59 (2.39)	0.50
Parity			0.55
0	23 (67.65)	22 (68.75)	
1	9 (26.47)	10 (31.25)	
2	2 (5.88)	0 (0)	
BMI, kg/m^2^	23.5* (4.13)	23.77* (4.24)	0.44
BMI category,			0.86
Underweight, < 18.5 kg/m^2^	1 (3.0)	3 (9.1)	
Normal weight, 18.5–22.9 kg/m^2^	17 (51.5)	17 (51.5)	
Overweight, 23.0–27.4 kg/m^2^	7 (21.2)	6 (18.2)	
Obese, > = 27.5 kg/m^2^	8 (24.2)	7 (21.2)	
Waist to hip ratio	0.85 (0.06)	0.86 (0.04)	0.60
HbA1c, mmol	33.2 (2.96)	32.5 (3.09)	0.33
Prior GDM, yes	0 (0)	0 (0)	–
OGTT			
Fasting glucose, mmol/L	4.91 (0.5)	4.8 (0.26)	0.16
2-h glucose, mmol/L	6.1 (1.61)	5.48 (1.37)	0.13
Prediabetes, yes	1 (3.23)	2 (6.25)	0.10
Family history of DM, yes	12 (36.36)	7 (21.88)	0.27
Time-to-pregnancy, months	2.45 (7.74)	3.84 (5.22)	0.86

*Abbreviations*: *GDM* Gestational diabetes mellitus, *IQR* Inter-quartile range, *BMI* Body mass index, *HbA1c* Glycated hemoglobin, *OGTT* Oral glucose tolerance test, *DM* Diabetes

^*^Accounted for matching with generalized linear mixed model or generalized estimating equation, if applicable

**Fig. 1:**
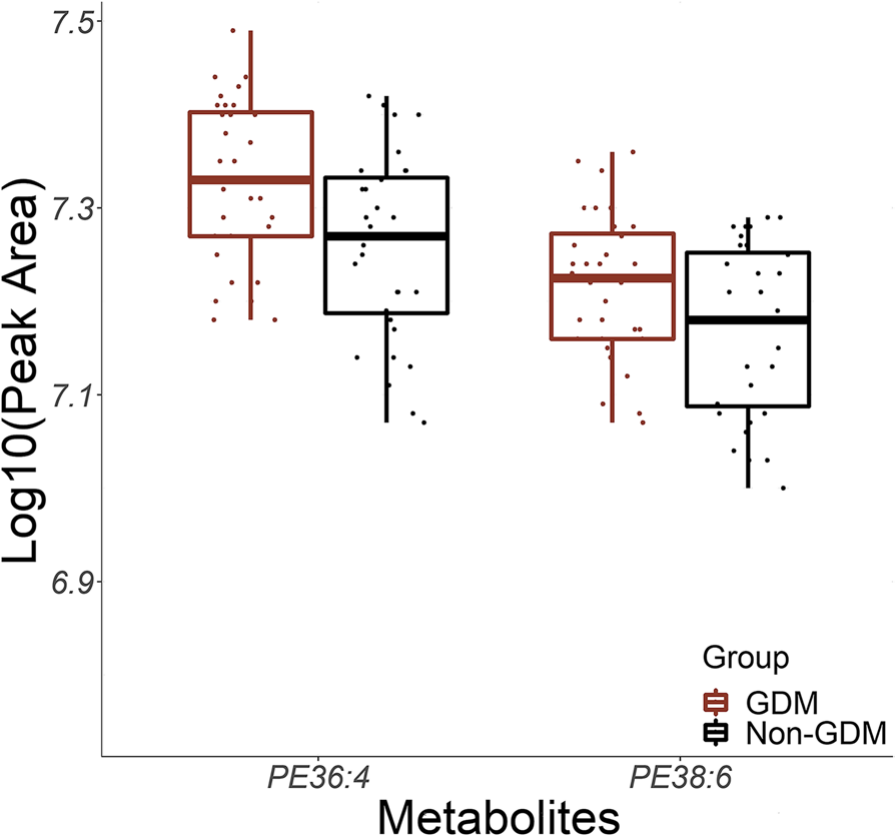
Scatter plots and box plots of phosphatidylethanolamines 36:4 and 38:6 at preconception (within 12 months prior to conception) phase between GDM and non-GDM controls in the nested case–control study embedded in SPRESTO study

These eight phosphatidylethanolamines expressed significantly higher signals in the GDM than the non-GDM controls (*β* range of mean difference: 0.04–0.07, all *p* < 0.05). The intra-CV in these eight phosphatidylethanolamines ranged between 2.86 and 4.14% (Additional file 2: Tab. S2). After FDR correction and sensitivity analysis, phosphatidylethanolamines 36:4 (mean difference *β*: 0.07; 95% CI: 0.02, 0.11, FDR-corrected *p*-value: 0.0084) and 38:6 (mean difference *β*: 0.06; 95% CI: 0.004, 0.11; FDR-corrected *p*-value: 0.0032) remained significantly different between GDM and non-GDM controls (Table 2) during preconception phase. We applied scatterplot overlaying box plots to showcase the distribution and signal mean differences between GDM and non-GDM controls according to eight phosphatidylethanolamines (Additional file 3: Fig. S1) and highlighted 36:4 and 38:6 in Fig. 1. Sensitivity analysis by additionally adjusting for family history of diabetes, parity, prediabetes during the preconception phase, and prior GDM history did not significantly attenuate our findings on 36:4 and 38:6 (Additional file 4: Tab.S3).

**Table 2:** Comparison of eight annotated metabolites between GDM and non-GDM subjects using GLMM during preconception phase within 12 months prior to conception

Phosphatidylethanolamines	GDM vs. non-GDM (reference)		
	Mean difference *β* (95% CI)	*p* value	FDR
34:1	0.04 (− 0.01, 0.09)	***0.04***	0.0498
34:2	0.06 (− 0.01, 0.12)	***0.03***	0.0498
36:2	0.05 (− 0.004, 0.11)	***0.049***	0.0498
36:4	0.07 (0.02, 0.11)	***0.001***	***0.0084***
38:4	0.05 (0.01, 0.09)	***0.03***	0.0498
38:5	0.04 (− 0.003, 0.09)	***0.04***	0.0498
38:6	0.06 (0.004, 0.11)	***0.01***	***0.0332***
40:6	0.06 (0.0002, 0.11)	***0.049***	0.0498

GLMM was adjusted for maternal age and pre-pregnancy body mass index (pp-BMI) at the preconception phase

*Abbreviations*: *GDM* Gestational diabetes mellitus, *GLMM* Generalized linear mixed model, *FDR* False discovery rate

Furthermore, no pathway was identified among eight annotated metabolites, whereas 12 pathways were identified among unannotated metabolites via KEGG pathway analysis (Additional file 5: Tab. S4).

**Fig. 2:**
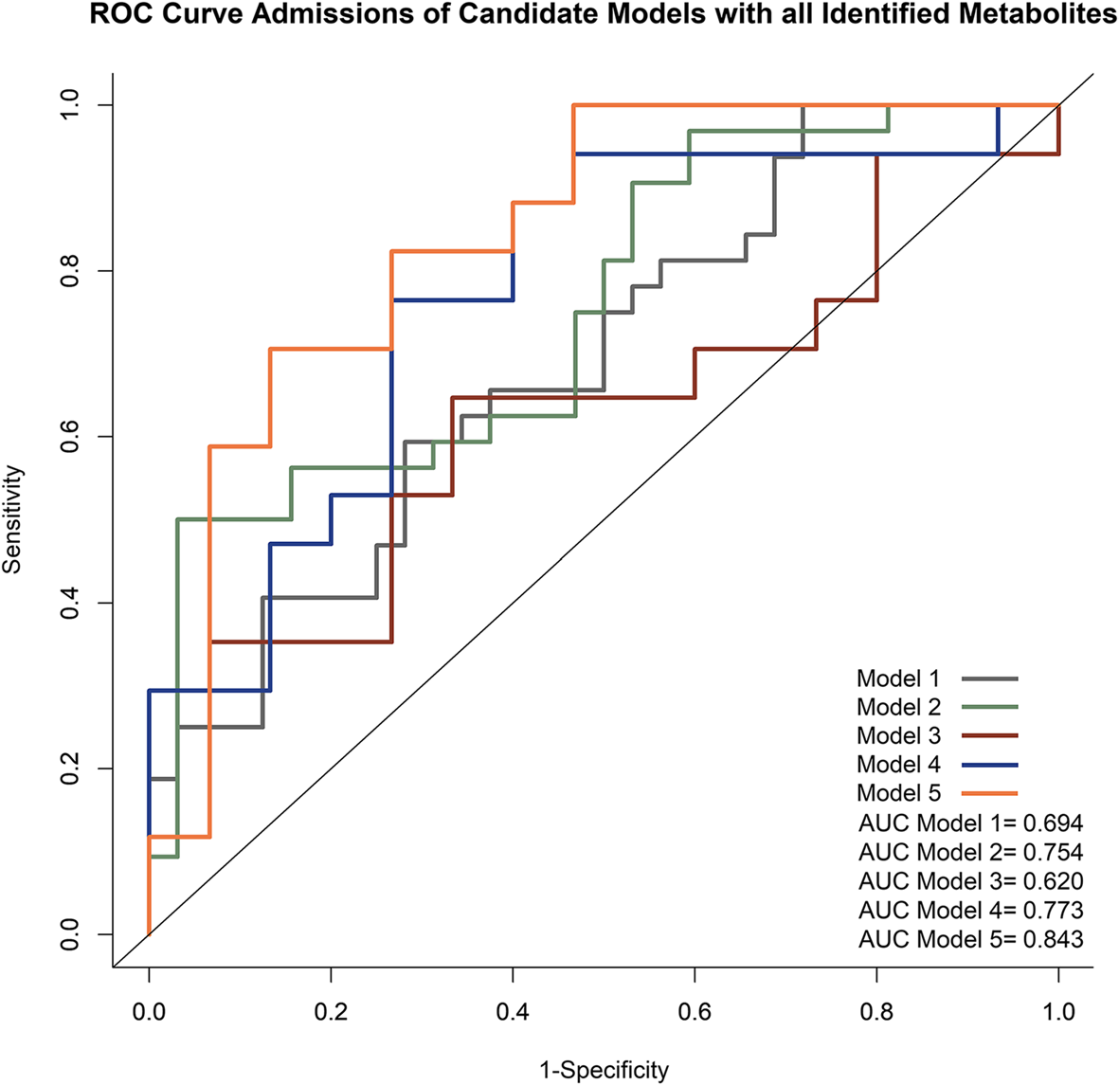
Receiver operating characteristic (ROC) curve admissions of the predictive models on GDM using identified metabolites and traditional risks. The red line represents the ROC curve of model 3: GDM ~ all traditional maternal risk factors including maternal age, ppBMI, family history of diabetes, prior history of GDM, preconception prediabetes and parity at the preconception phase (*R*^2^ = 0.048, AUC = 0.620). The yellow line represents the ROC curve of model 5: GDM ~ all eight metabolites identified at preconception in addition to model 3 (*R*^2^ = 0.377, AUC = 0.843)

Since there were eight metabolites identified while two remained significant after FDR correction, we performed the ROC curve for all candidate models based on different sets of metabolites. With 36:4 and 38:6 signals in addition to traditional risk factors such as maternal age, ppBMI, family history of diabetes, prior history of GDM, preconception prediabetes, and parity, the AUC increased from 0.620 to 0.773 and *R*^2^ increased from 0.048 to 0.236 (Table 3 and Fig. 2). With all eight metabolites’ signals in addition to the same set of traditional risk factors mentioned above, the AUC increased from 0.620 to 0.843 and *R*^2^ increased from 0.048 to 0.377 (Table 3 and Fig. 2). Due to the multiple adjustments within a relatively small sample size, all comparisons did not reach statistical significance.

**Table 3:** Predictive value for GDM using traditional and novel biomarkers identified in our cohort

Models	*R*^2^	AUC	*P* value		
			Ref (model 1)	Ref (model 2)	Ref (model 3)
Metabolites prediction model					
Model 1, adjusting for 36:4, 38:6	0.127	0.694	N/A	N/A	N/A
Model 2, adjusting for eight metabolites (34:1, 34:2, 36:2, 36:4, 38:4, 38:5, 38:6, and 40:6)	0.194	0.754	0.554	N/A	N/A
Traditional risks prediction model					
Model 3, adjusting for maternal age, ppBMI, family history of GDM, prior history of GDM, preconception prediabetes, parity	0.048	0.620	0.382	0.612	N/A
Metabolites and traditional risks combined prediction model					
Model 4, model 1 + model 3	0.236	0.773	0.360	0.738	0.480
Model 5, model 2 + model 3	0.377	0.843	0.433	0.257	0.300

*Abbreviations*: *GDM* Gestational diabetes mellitus, *ppBMI* pre-pregnancy body mass index, *AUC* Area under the curve

## Discussion

In our longitudinal study of 33 pairs of Chinese GDM and non-GDM controls on non-targeted metabolomics signature at the preconception phase within 12 months prior to conception, fifty-seven metabolites were significantly related to GDM. Among them, eight phosphatidylethanolamines were successfully annotated with a range of fatty acid chain lengths. After FDR correction in multiple testing, only phosphatidylethanolamines 36:4 and 38:6 remained significant in association with GDM. Compared with non-GDM controls, these two glycerophospholipids were related to adverse cardiometabolic profiles and exhibited significantly higher signals during the preconception phase in GDM subjects.

Emerging evidence has shown that serum, plasma, and even urine metabolites (e.g., lipids, fatty acids, amino acids, acylcarnitines, dopamine) were associated with incident GDM in either early or mid-pregnancy [8–11, 23, 24]. However, we are unaware of studies on metabolomics measurements before pregnancy. Women at risk of GDM already exert differences in fat deposition and glycose tolerance even before their pregnancy [25]. The signatures of metabolites in preconception are of potential value to understand the pathophysiology of GDM.

Our study is the first to investigate the missing link of changes in metabolites as early as in the preconception phase. Among the eight metabolites identified to differentiate GDM from non-GDM controls, all were phosphatidylethanolamines—a class of glycerophospholipids that is made in the endoplasmic reticulum (ER) via the cytidine diphosphate-diacylglycerol-ethanolamine pathway [26]. Since phosphatidylethanolamine is one of the most abundant glycerophospholipids in mammalian cells and is easy to obtain from human blood and small biopsy tissues, clinical studies in the past decade have widely investigated its association with insulin sensitivity. Emerging evidence has demonstrated the key role of phosphatidylethanolamine in the insulin signaling pathway, and it was suggested that increased phosphatidylcholine/phosphatidylethanolamines ratio was associated with reduced insulin sensitivity [27] and elevated among patients with type 2 diabetes [28]. In mice models, accumulation of phosphatidylethanolamine production in mitochondria is suggested to modulate glucose [29] and increase diacylglycerol [30], the latter of which is known for causing insulin resistance in cells [31].

Our study showed a significantly higher signal of phosphatidylethanolamines 36:4 and 38:6 in GDM cases than in non-GDM controls. Emerging evidence also showed elevated levels of phosphatidylethanolamines (e.g., 18:1, 22:2, 36:1, 36:4, and 38:6) in both mid- and late pregnancy [11, 32], among women with different racial backgrounds. Even though their biological functions underlying the pathogenesis of GDM are largely unknown, we postulated that phosphatidylethanolamines could adversely impact cellular activity and glucose and fatty acid metabolism [27], since lipid metabolism disorders often accompany glucose metabolism disorders in diabetes, and the complex relationship between metabolism and numerous lipid metabolites needs further elucidation. A recent longitudinal study also reported that glycerophospholipids could predict the transition from GDM to type 2 diabetes in the early postpartum period, which was a superior indicator to clinical parameters [33]. Our findings and others might provide strong evidence to pinpoint the consistent and distinctive values of certain types of phosphatidylethanolamines related to GDM, from preconception to postpartum phases. And the identification of these might help classify and prevent GDM and even postpartum type 2 diabetes among women at risk. Future studies should explore such metabolites and pathways underlying the GDM etiology in alternative populations and with larger sample sizes.

The strength of this study lies in the preconception blood sample within 12 months prior to conception, and the comprehensive measures of metabolomics based on an untargeted approach, from a group of homogenous Chinese women. The study is not without limitations. Firstly, the relatively small sample size may limit the statistical power of the study. We are not able to validate our results in a subset of samples within our cohort. However, even with 33 pairs of GDM cases and controls, we robustly annotated two metabolites that could distinctively differentiate GDM subjects from non-GDM controls after FDR correction. Secondly, the study design of one-time measurement of metabolites within 12 months prior to conception might not capture the dynamic trajectories of metabolic profiles. Even though it may be practically much more challenging, future studies with longitudinal measures before pregnancy are warranted. Considering our subjects were more motivated to maintain a relatively healthy lifestyle and physique to achieve a successful pregnancy than the general population [34], and those who entered pregnancy with livebirth outcomes had a more healthful plant-based eating dietary pattern [35], the impact of identifying metabolites due to dynamic trajectories in our study is speculated less significant than the general population. Lastly, even though we developed the prediction model from a nested case–control study, the under-sampling of non-outcomes might potentially overestimate the AUC performance in our study.

## Conclusions

Our data identified distinctive signatures of metabolites of GDM, specifically in preconception fasting serum (i.e., phosphatidylethanolamines 36:4 and 38:6), which is of potential value to understand in depth on the etiology of GDM as early as in the preconception phase. Future studies with larger sample sizes in a multiracial prospective study setting with external validation and multiple time points of metabolites testing are warranted to validate the association of these signatures with GDM and even evaluate the predictive value of such metabolites.

## Supplementary Information

**Additional file 1:** **Tab. S1.** Spearman rank correlation among all annotated phosphatidylethanolamines (*n* = 8).

**Additional file 2:** **Tab. S2.** The intra-coefficient of variation (CV) among all identified phosphatidylethanolamines in our study after eight attempts of quality control.

**Additional file 3:** **Fig. S1.** Scatter plots and box plots of eight annotated phosphatidylethanolamines at preconception (within 12 months prior to conception) phase between GDM and non-GDM controls in the nested case–control study embedded in SPRESTO study.

**Additional file 4:** **Tab S3.** Sensitivity analysis.

**Additional file 5:** **Tab. S4.** KEGG pathway analyses of all GLMM-identified and yet unannotated metabolites (*n* = 48).

## Data Availability

Data will be made available to the editors of the journal for review or query upon request. Individual participant data (including data dictionaries) underlie the results reported in this article after deidentification will be available if an investigator who is an independent review committee from SPRESTO cohort has approved the proposed use of the data identified for the research purpose. The data are available between 9 and 36 months following article publication.

## Abbreviations

GDM: Gestational diabetes mellitus
SPRESTO: Singapore PREconception Study of long-Term maternal and child Outcomes
IADPSG: International Association of Diabetes and Pregnancy Study
ppBMI: Pre-pregnancy body mass index
LC-MS: Liquid chromatography-mass spectrometry
CV: Coefficient of variation
OGTT: Oral glucose tolerance test
WHO: World Health Organization
GLMM: Generalized linear mixed model
GEE: Generalized estimation equation
HMDB: Human Metabolite Database
FDR: False discovery rate
IQR: Inter-quartile range
ROC: Receiver operating characteristic
AUC: Area under the curve
SD: Standard deviation
ER: Endoplasmic reticulum
