# Supplementary material for: Exploring preconception signatures of metabolites in mothers with gestational diabetes mellitus using a non-targeted approach
Source: BMC Med. 2023 Mar 16;21:99. doi: 10.1186/s12916-023-02819-5 (PMC10022116; doi:10.1186/s12916-023-02819-5)
Supplement: Supplementary file 1 — Additional file 1: Tab. S1. Spearman rank correlation among all annotated phosphatidylethanolamines (n = 8). [file 12916_2023_2819_MOESM1_ESM.docx]

**Additional file 1: Tab. S1. Spearman rank correlation among all annotated phosphatidylethanolamines (n=8)**

|  | 34:2 | 34:1 | 36:4 | 36:2 | 38:6 | 38:5 | 38:4 | 40:6 |
| --- | --- | --- | --- | --- | --- | --- | --- | --- |
| 34:2 | 1 |  |  |  |  |  |  |  |
| 34:1 | 0.82  (p<0.001) | 1 |  |  |  |  |  |  |
| 36:4 | 0.51  (p<0.001) | 0.64  (p<0.001) | 1 |  |  |  |  |  |
| 36:2 | 0.91  (p<0.001) | 0.83  (p<0.001) | 0.52  (p<0.001) | 1 |  |  |  |  |
| 38:6 | 0.30  (p=0.01) | 0.54  (p<0.001) | 0.69  (p<0.001) | 0.41  (p<0.001) | 1 |  |  |  |
| 38:5 | 0.40  (p<0.001) | 0.59  (p<0.001) | 0.83  (p<0.001) | 0.52  (p<0.001) | 0.7  (p<0.001) | 1 |  |  |
| 38:4 | 0.40  (p<0.001) | 0.53  (p<0.001) | 0.86  (p<0.001) | 0.54  (p<0.001) | 0.67  (p<0.001) | 0.87  (p<0.001) | 1 |  |
| 40:6 | 0.26  (p<0.001) | 0.45  (p<0.001) | 0.56  (p<0.001) | 0.50  (p<0.001) | 0.80  (p<0.001) | 0.69  (p<0.001) | 0.78  (p<0.001) | 1 |
