# Supplementary material for: Exploring preconception signatures of metabolites in mothers with gestational diabetes mellitus using a non-targeted approach
Source: BMC Med. 2023 Mar 16;21:99. doi: 10.1186/s12916-023-02819-5 (PMC10022116; doi:10.1186/s12916-023-02819-5)
Supplement: Supplementary file 2 — Additional file 2: Tab. S2. The intra-coefficient of variation (CV) among all identified phosphatidylethanolamines in our study after eight attempts of quality control. [file 12916_2023_2819_MOESM2_ESM.docx]

**Additional file 2: Tab. S2**. **The intra-coefficient of variation (CV) among all identified phosphatidylethanolamines in our study after eight attempts of quality control.**

|  | 38:6 | 38:5 | 34:2 | 36:4 | 40:6 | 34:1 | 38:4 | 36:2 |
| --- | --- | --- | --- | --- | --- | --- | --- | --- |
| Mean | 16329268 | 12324979 | 3344110 | 20844787 | 12460045 | 1535434 | 26770418 | 7659084 |
| SD | 466320 | 354755 | 128152 | 626792 | 479169 | 63567 | 778368 | 251238 |
| Intra-CV(%) | 2.86 | 2.88 | 3.83 | 3.01 | 3.85 | 4.14 | 2.91 | 3.28 |

Abbreviations: CV, coefficient of variation; SD, standard deviation.
