# Supplementary figures and images for: Exploring preconception signatures of metabolites in mothers with gestational diabetes mellitus using a non-targeted approach
Source: BMC Med. 2023 Mar 16;21:99. doi: 10.1186/s12916-023-02819-5 (PMC10022116; doi:10.1186/s12916-023-02819-5)

Log10(Peak Area)

PE34:1

PE34:2

PE36:2

PE36:4

PE38:4

PE38:5

PE38:6

PE40:6

Metabolites

Group

GDM

Non-GDM

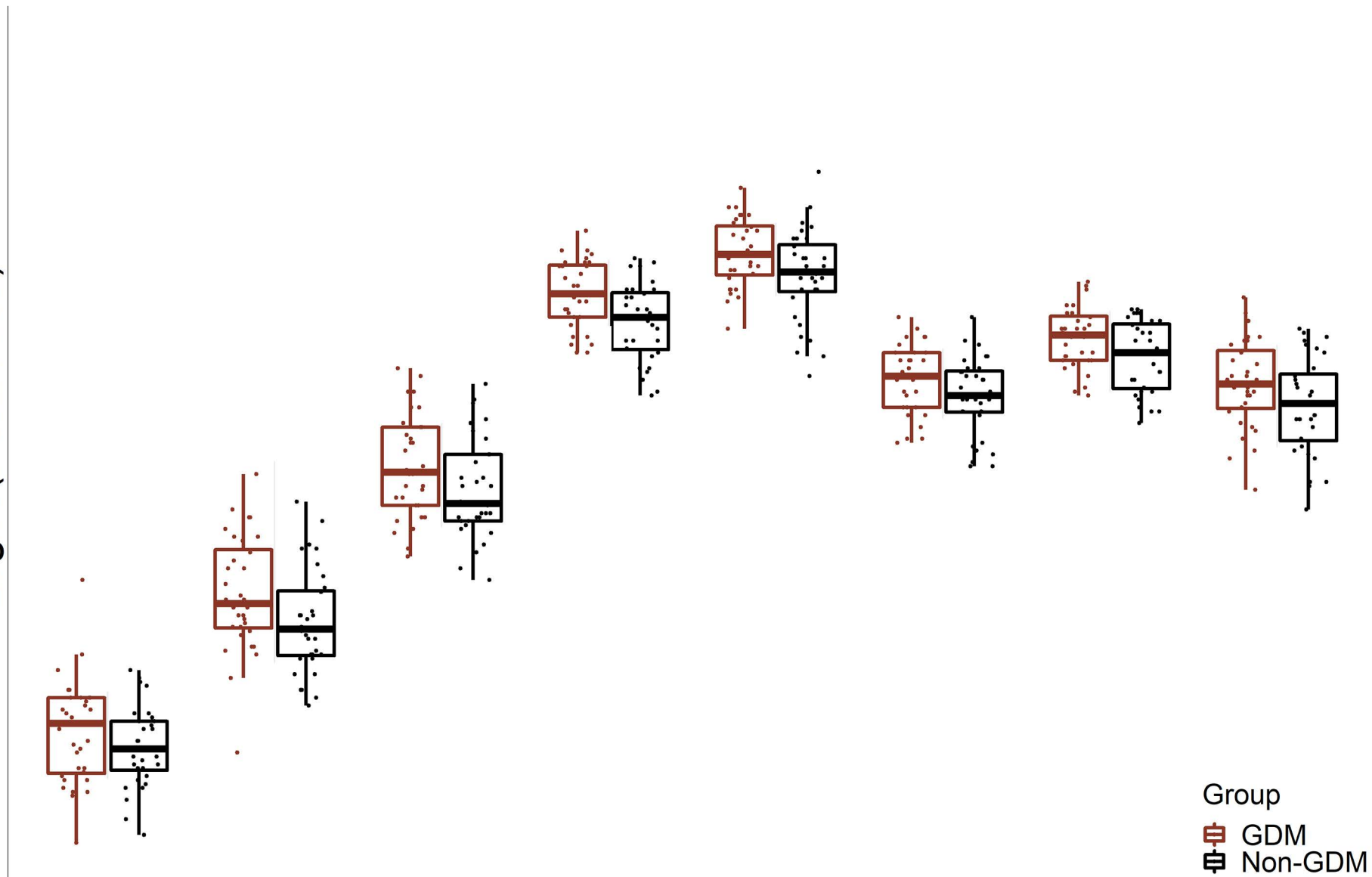

Supplement: Supplementary file 3 — Additional file 3: Fig. S1. Scatter plots and box plots of eight annotated phosphatidylethanolamines at preconception (within 12 months prior to conception) phase between GDM and non-GDM controls in the nested case–control study embedded in SPRESTO study. [file 12916_2023_2819_MOESM3_ESM.pdf]
