# Supplementary material for: Exploring preconception signatures of metabolites in mothers with gestational diabetes mellitus using a non-targeted approach
Source: BMC Med. 2023 Mar 16;21:99. doi: 10.1186/s12916-023-02819-5 (PMC10022116; doi:10.1186/s12916-023-02819-5)
Supplement: Supplementary file 4 — Additional file 4: Tab S3. Sensitivity analysis. [file 12916_2023_2819_MOESM4_ESM.docx]

**Additional file 4: Tab S3. Sensitivity analysis**

| **phosphatidylethanolamines** | **GDM vs. non-GDM (reference)** | |
| --- | --- | --- |
|  | Mean difference  β (95% CI) | P value |
| Adjusting for maternal age and ppBMI | | |
| 36:4 | 0.07 (0.02, 0.11) | ***0.001*** |
| 38:6 | 0.06 (0.004, 0.11) | ***0.01*** |
| Adjusting for maternal age, ppBMI and family history of diabetes | | |
| 36:4 | 0.07 (0.03, 0.12) | ***0.003*** |
| 38:6 | 0.05 (0.01, 0.09) | ***0.03*** |
| Adjusting for maternal age, ppBMI and parity | | |
| 36:4 | 0.07 (0.03, 0.12) | ***0.003*** |
| 38:6 | 0.05 (0.01, 0.09) | ***0.02*** |
| Adjusting for maternal age, ppBMI and pre-pregnancy prediabetes | | |
| 36:4 | 0.07 (0.03, 0.11) | ***0.004*** |
| 38:6 | 0.05 (0.01, 0.09) | ***0.02*** |
| Adjusting for maternal age, ppBMI and prior GDM history | | |
| 36:4 | 0.07 (0.03, 0.11) | ***0.004*** |
| 38:6 | 0.05 (0.01, 0.09) | ***0.02*** |
| Adjusting for maternal age, ppBMI and time-to-pregnancy | | |
| 36:4 | 0.07 (0.01, 0.09) | ***0.01*** |
| 38:6 | 0.05 (0.01, 0.09) | ***0.03*** |

Abbreviations: GDM, gestational diabetes mellitus; ppBMI, pre-pregnancy body mass index
