# Supplementary material for: Exploring preconception signatures of metabolites in mothers with gestational diabetes mellitus using a non-targeted approach
Source: BMC Med. 2023 Mar 16;21:99. doi: 10.1186/s12916-023-02819-5 (PMC10022116; doi:10.1186/s12916-023-02819-5)
Supplement: Supplementary file 5 — Additional file 5: Tab. S4. KEGG pathway analyses of all GLMM-identified and yet unannotated metabolites (n = 48). [file 12916_2023_2819_MOESM5_ESM.docx]

**Additional file 5: Tab. S4. KEGG pathway analyses of all GLMM-identified and yet unannotated metabolites (n=48)**

| **Pathway Name** | **KEGG ID** | **Total Hits** | **Significant Hits** | **P value** | **Compound Hits m/z** | **Annotated in this study** |
| --- | --- | --- | --- | --- | --- | --- |
| Arginine biosynthesis | map00220 | 1 | 1 | 0.15 | 177.1041 | No |
| Galactose metabolism | map00052 | 2 | 1 | 0.28 | 384.1509 | No |
| Arginine and proline metabolism | map00330 | 2 | 1 | 0.28 | 207.997 | No |
| Tryptophan metabolism | map00380 | 2 | 1 | 0.28 | 103.1021 | No |
| Mannose type O-glycan biosynthesis | map00515 | 2 | 1 | 0.28 | 791.5823 | No |
| Glycosylphosphatidylinositol (GPI)-anchor biosynthesis | map00563 | 3 | 1 | 0.39 | 410.2964 | No |
| Lysine degradation | map00310 | 4 | 1 | 0.48 | 209.9946 | No |
| Drug metabolism - cytochrome P450 | map00982 | 11 | 2 | 0.51 | 271.9935; 312.1508 | No |
| Metabolism of xenobiotics by cytochrome P450 | map00980 | 5 | 1 | 0.56 | 312.1508; 228.1156 | No |
| Steroid hormone biosynthesis | map00140 | 19 | 2 | 0.82 | 174.1151; 273.1853 | No |
| Primary bile acid biosynthesis | map00120 | 16 | 1 | 0.93 | 410.2964 | No |
| Steroid biosynthesis | map00100 | 25 | 1 | 0.99 | 387.3428 | No |

Abbreviations: GLMM, generalized linear mixed model.
